# Stakeholder perspectives of barriers and facilitators to enhancing hydration in elementary schools within a socio-ecological framework

**DOI:** 10.3389/fpubh.2026.1784617

**Published:** 2026-04-22

**Authors:** Kristina L. Tatum, Danyel I. Smith, Hsinling Sonya Hung, Madison Weinstock, Shannon Rhodenhiser, Justine Blincoe, Alisa E. Brewer, Jessica Gokee LaRose, Tisha Erby, LaTriece Haskins, Kevin Starlings, Katlyn Garr, Tegwyn H. Brickhouse, Melanie K. Bean, Hydration Community Advisory Board

**Affiliations:** 1 Department of Pediatrics, Children’s Hospital of Richmond at Virginia Commonwealth University, Richmond, VA, United States; 2 Department of Social and Behavioral Sciences, School of Public Health, Virginia Commonwealth University, Richmond, VA, United States; 3 Department of Oncology, Georgetown University Medical Center and Cancer Prevention and Control Program, Georgetown-Lombardi Comprehensive Cancer Center, Washington, DC, United States; 4 Center on Health Advancement, School of Medicine, Virginia Commonwealth University, Richmond, VA, United States; 5 Greater Richmond Fit4Kids, Richmond, VA, United States; 6 Hydration Community Advisory Board; 7 The Starlings Foundation, Richmond, VA, United States; 8 Department of Dental Public Health & Policy, School of Dentistry, Virginia Commonwealth University, Richmond, VA, United States

**Keywords:** children, community engagement, drinking water, elementary schools, hydration

## Abstract

**Introduction:**

Adequate hydration is vital for cognitive performance, fine motor skills, and visual attention, foundational for academic success. However, children spend a considerable portion of their waking hours at school, where they do not consume enough water. The Healthy, Hunger-Free Kids Act of 2010 requires school district wellness policies to implement provisions ensuring that free, clean, accessible, safe drinking water is available during the school day, including mealtimes. Despite ongoing policy efforts, numerous barriers to equitable drinking water access persist, particularly in lower-resourced school districts. This study explores stakeholders’ perspectives on barriers and facilitators influencing the hydration landscape in an urban school district in central Virginia to inform the development of a school-based intervention.

**Methods:**

A community-engaged qualitative approach was employed, with semi-structured interviews (*N*=15) conducted among wellness leaders, school personnel, and site coordinators. Trained coders applied a multi-step grounded theory approach to identify themes across multiple levels of the social-ecological model. A multi-sectorial community advisory board contributed to the design, interview guide questions, and interpretation of findings.

**Results:**

Stakeholders identified nine key themes: (1) perceived importance of hydration (e.g., health benefits, water as a necessity); (2) students’ motivation to drink water (e.g., hydration station novelty, perceived water source cleanliness); (3) teachers’ role in student hydration (e.g., role modeling, providing education on healthy hydration); (4) home environment influences (e.g., available hydration options, water quality); (5) classroom management strategies (e.g., hydration breaks); (6) access to resources (e.g., availability of reusable water bottles); (7) competing school priorities (e.g., teacher workload, standardized testing); (8) community influences (e.g., access to healthy hydration options); and (9) awareness of current hydration policies (e.g., communicating policy efforts).

**Discussion:**

Stakeholders identified barriers and facilitators influencing hydration in schools. Access to resources, community partnerships, and classroom hydration policies, emerged as key intervention targets for achieving water equity in elementary schools.

## 1 Introduction

Suboptimal hydration is a persistent barrier to child health (1). Inadequate water intake is linked to poor oral health (e.g., dental caries), challenges with weight maintenance, and diminished academic outcomes (1–4). Developmentally, children have substantial physiological needs for adequate hydration, due to their developing kidneys and digestive systems (1). Academically, water supports cognitive performance, including attention, coordination, and visual processing, which are important functions for learning (4–6). Despite the benefits of hydration, inadequate access to safe, potable water often hinders sufficient water intake among children and adolescents (7). Schools serve as a central, structured context for promoting healthy hydration. The Healthy, Hunger-Free Kids Act (HHFKA) of 2010 requires schools to develop and implement wellness policies, including mandating that potable water is freely available during school hours (8). However, federal mandates alone have not yielded optimal water access and uptake among children, due to varying district- and school-level constraints and unique student population needs. Each school district has unique strengths and barriers. Improving the impact and sustainability of hydration efforts require a community-engaged approach that leverages stakeholders’ strengths, builds capacity, and strategically district- and school-level barriers.

Title I schools receive federal funding to support children from low-income families (9, 10). These federally funded schools often face infrastructure and resource-related challenges and predominantly serve Black and Latino/a/x student populations (11, 12). Many of these schools have aging infrastructure, including lead pipes, outdated water fountain/sources, and are generally lower resourced (13). These conditions further exacerbate hydration disparities and delay the implementation of school-based water-policies. Moreover, the visible deterioration of drinking water infrastructure and past incidents of water contamination (e.g., lead), negatively impact perceptions of water safety and cleanliness among the school community. These concerns among school personnel, and parents ultimately hinder water intake during school hours (12, 13). We previously partnered with a local non-profit organization and the local health district to improve hydration within an urban school district in central Virginia (VA). The majority of the elementary schools in this district are designated as Title I (14). These initial efforts included installing a single hydration station (i.e., water bottle refill station), distributing reusable water bottles, and conducting a one-week water promotion campaign. This short-term intervention resulted in an initial increase in water use post-water promotion activities, a reduction in soda consumption, and an increase in a self-reported use of the hydration station (14). However, following those initial efforts, hydration stations were removed due to the detection of lead in the school’s infrastructure, which has since been addressed.

Recently, the School Health Advisory Board renewed its commitment to optimizing hydration in the district. In response, the district identified the need for additional efforts to optimize water intake within this policy and environment. We established a Community Advisory Board (CAB) of key stakeholders and used a participatory approach (9) to assess community needs and strengths related to hydration. Consultations with teachers and CAB members highlighted both the successes and challenges within the policy. We further leveraged our academic-community partnerships to conduct a comprehensive environmental scan of the hydration landscape. This formative work aimed to: (1) identify strengths and capacity-building needs and (2) inform the development of a school-based, community-participatory hydration intervention.

### 1.1 Theoretical framework

Grounded in Bronfenbrenner’s socio-ecological model (15, 16), the socio-ecological model (SEM) posits that behaviors are shaped by the dynamic interplay of individual and environmental factors. In the context of water equity, this framework can elucidate interrelated systems, including individual, interpersonal, institutional, community, and policy level systems that influence water intake. The SEM has been broadly and successfully applied to other formative research in school-based health promotion (17–19).

Stakeholders whom are embedded within different levels of a community (e.g., school classroom, community organizations) can offer critical insight into multilevel barriers to school-based hydration efforts. Their involvement improves the relevance and sustainability of these efforts. Moreover, community/academic partnerships and advisory boards bolster the development of evidence-based, sustainable, multilevel interventions. Such collaborations are essential to build school capacity, promote healthy hydration, and foster a school culture of health (14). Prior work has applied the SEM as a guiding framework for these type of community engaged initiatives (10).

However, few studies have applied the SEM within formative research to specifically examine water access and consumption in underserved elementary schools using a community-engaged approach. For example, some studies have examined hydration without a multilevel theoretical framework (20), others applied the SEM across multiple school levels without capturing diverse community stakeholder perspectives (10, 21), or utilized related ecological frameworks in different cultural context without addressing equity considerations specific to underserved schools (21). To our knowledge, no prior studies have applied the SEM using a community-engaged approach to examine multilevel hydration barriers and facilitators within urban Title I elementary schools. In these settings, infrastructure inequities, resource constraints, and policy implementation gaps create unique challenges for promoting student hydration.

In collaboration with our CAB, the present study addresses this gap by conducting semi-structured qualitative interviews to preemptively explore the perspectives of diverse stakeholders with both direct and indirect roles in an urban Title I elementary school district. This exploration utilizes the SEM framework to inform our understanding of the multi-level barriers and facilitators influencing the school hydration landscape. Findings will inform a needs- and asset-based, viable health promotion school intervention designed to advance equitable water access and enhance healthy hydration practices in elementary schools.

## 2 Materials and methods

### 2.1 Study design

A community-engaged qualitative approach was used to explore stakeholders’ perceptions of school hydration. Individual semi-structured interviews assessed needs, barriers, challenges, and assets related to healthy hydration in an urban school district in central Virginia. The Consolidated Criteria for Reporting Qualitative Studies (COREQ) guided data collection and reporting (22). This study was part of a larger project supported by a multi-sectorial CAB composed of eight members representing diverse stakeholder groups (i.e., students, parents, teachers, and school nutrition personnel). We partnered with the CAB throughout the research process, including the development of interview guide questions, identification of stakeholder groups, participant recruitment, interpretation of findings, and data dissemination. Study procedures were approved by Virginia Commonwealth University Institutional Review Board.

### 2.2 Participants and recruitment

This study was conducted in an urban school district in central Virginia serving approximately 10,300 K-5 students across 25 elementary schools, of which 23 are designated as Title I schools, with key informants selected to represent the district as a whole, rather than representing “schools,” and provide perspectives across three school community categories. The district’s student population includes students who identify primarily as Black (58%), Latino/a/x (25%), and White (13%). The majority (>90%) of students participate in the National School Lunch Program, and all students receive free meals through the Community Eligibility Provision (23).

With guidance from the CAB, stakeholders were recruited through existing community partners and networks within one urban school district in central Virginia. Participants were initially selected using purposive sampling to ensure representation across the following three school community categories: (1) wellness leaders (e.g., individuals external to the school’s daily operations), (2) school personnel (e.g., individuals internal to the school’s daily operations), and (3) site coordinators (e.g., individuals working inside the school, but employed by outside organizations). A snowball sampling approach (e.g., leveraging existing participants’ network) was used to identify additional stakeholders within each category.

Potential participants (*n* = 27) received an email describing the study which included a link to an online interest form administered through REDCap (electronic data capture tools hosted at Virginia Commonwealth University) (24). Eligible respondents were contacted by study personnel via email to schedule a virtual interview, with recruitment continuing until 15 interviews [the *a priori* target sample size established based on published guidance on achieving saturation in qualitative research (25, 26)], representing the three school community categories, were completed. Data saturation was confirmed at this sample size, when no new theme emerged.

### 2.3 Interview guide and methods

An interview protocol was developed with topics, questions, and probes derived from the literature on current hydration policies (i.e., HHFKA of 2010) (27) and findings from previous school-based needs assessments that identified specific barriers to student hydration. Questions and probes were refined in collaboration with our CAB. Interview domains included the following: participant role in student hydration; perceived barriers and facilitators of healthy hydration, existing district-level policies, efforts related to student hydration; and recommendations to improve student hydration (see interview guide domains and questions in Appendix A). Semi-structured interviews were conducted via the video conferencing platform Zoom by trained study personnel (K. T., a postdoctoral fellow and D. S., a doctoral candidate) between May 2022 and October 2022. Interested participants provided electronic informed consent via REDCap and received a $25 gift card upon interview completion.

Interview duration was a range of 44–78 min. Stakeholders (*N* = 15) included: wellness leaders (e.g., school health advisory board members, Health and Physical Education lead); school personnel (e.g., classroom teachers, facilities managers, food service workers); and site coordinators [e.g., Communities in Schools (CIS) coordinators] (Table 1).

**Table 1:** Sample characteristics of stakeholders (*N* = 15).

Characteristics	*n* (%)
Gender	
Female	13 (86.7)
Male	2 (13.3)
Categories of stakeholders	
Wellness leaders	4 (26.7)
School personnel	4 (26.7)
Site coordinators	7 (46.7)

### 2.4 Data analysis

Interviews were audio recorded and transcribed verbatim via Zoom. Transcripts were cleaned by study staff to correct grammar, denote inaudible words, remove filler words, and redact personal identifiers prior to data analysis (28). To enhance scientific rigor and data integrity, we employed member checking by emailing transcripts to participants for review of accuracy. No modifications were suggested from participants. During the initial phase of thematic analysis, D. S. and K. T. read through a subset of the transcripts to familiarize themselves with the data. A preliminary coding structure was developed based on study domains, existing literature, and the SEM framework, reflecting a hybrid deductive-inductive analytic approach. The SEM framework deductively provided a foundational structure to guide the initial coding process. Using an inductive approach, the codebook was iteratively refined during team meetings as novel themes emerged from the data, ensuring a comprehensive understanding of stakeholders’ perspectives.

Transcripts were uploaded to Atlas.ti qualitative analysis software (28). Four trained coders (K. T., S. R., D. S., and M. W.), under the supervision of H. H. (Assistant Professor), used a multi-step grounded theory approach (29). First, the codebook was refined by independently reviewing a subset of the same transcripts to identify relevant codes and to discuss nuances and discrepancies. Second, coders independently analyzed a subset of transcripts, which initially yielded a moderate inter-coder agreement (*α* = 0.5), then a higher inter-coder agreement (*α* = 0.6) after discussing discrepancies. After finalizing the codebook, coders analyzed 3–4 transcripts independently. Any further discrepancies were discussed during weekly meetings. Coders utilized memos to document noteworthy connections within transcripts.

## 3 Results

**Figure 1:**
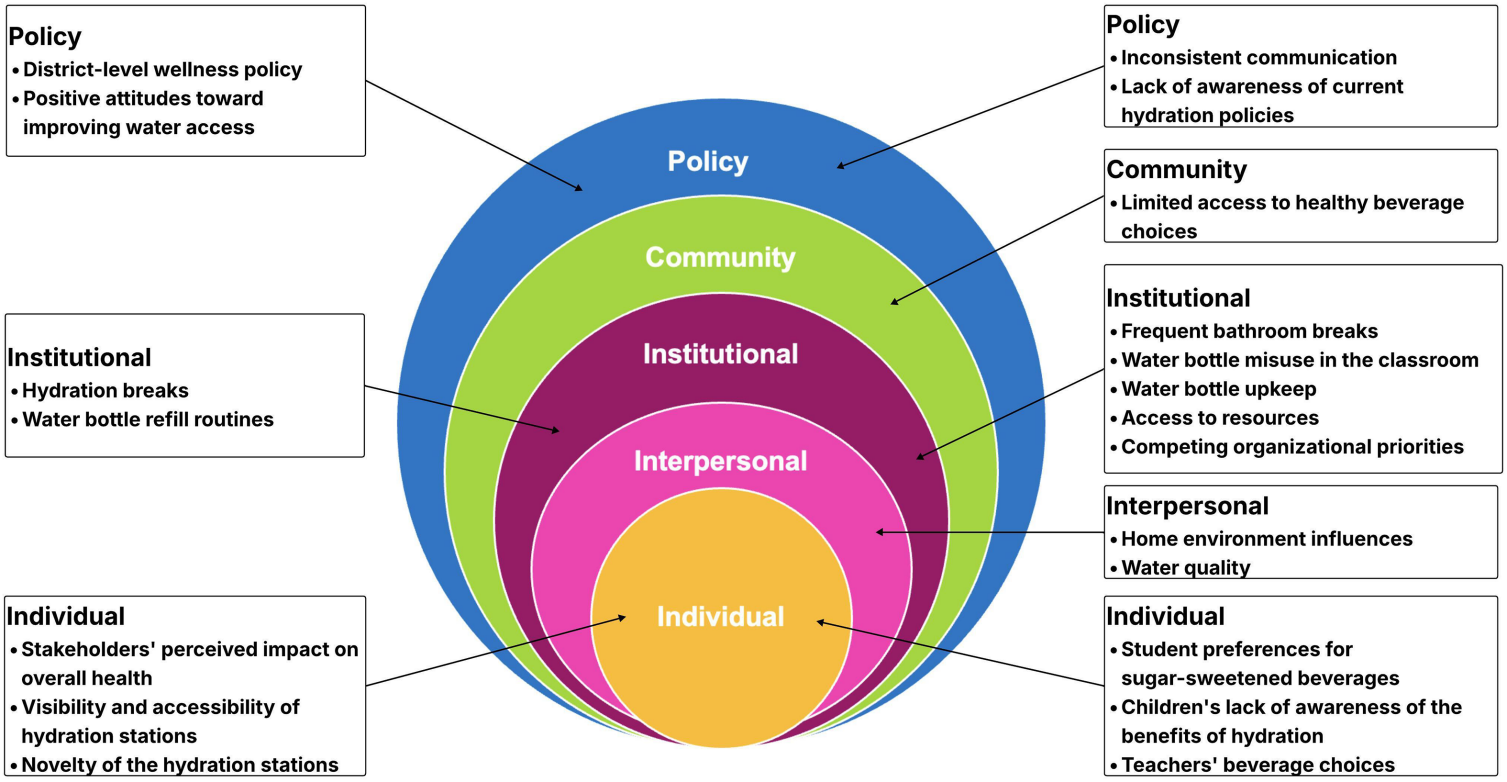
Barriers and facilitators of healthy hydration in elementary school students using the socio-ecological model.

Stakeholders shared overlapping perspectives on both policy and hydration efforts within the school, as well as their responsibility for improving student hydration. Barriers and facilitators emerged at the individual, interpersonal, institutional, community, and policy levels aligning with the SEM framework (15). Nine overarching themes, along with illustrative quotes are presented below and in Figure 1, organized by SEM level.

### 3.1 Individual level

#### 3.1.1 Perceived importance of hydration

Stakeholders emphasized the importance of hydration for children’s overall health, particularly its benefits to their mental health, physical functioning, cognitive performance, and general well-being. Several noted that water is a basic necessity and should be accessible throughout the school day.

*“If you don’t get enough fluids and you’re not hydrated, you’re less likely to be able to feel good and learn”* (School Personnel, P12).

*“I think [water is] an urgent need…we have kids that play sports. We have kids that are in classes and testing, and it’s a necessity…to drink water, because how do you function? It’s that important to me”* (Wellness Leader, P16).

#### 3.1.2 Students’ motivation to drink water

Despite stakeholders’ attitudes towards the necessity for healthy hydration, students’ motivation could be shaped by their interaction with their physical and social environment that contribute to the selection of other beverages. For example, stakeholders observed how the visibility and accessibility of the hydration stations facilitated healthy hydration behaviors among students:

“*From my perspective, from what I’m seeing, they’re getting a lot of hydration. All the kids like the fact, that they are carrying the water bottles around, [they’re] drinking more water, it’s good. I’m seeing that”* (School Personnel, P19).

The perceived taste of water was also identified as a factor influencing student’s decision to drink from the hydration station.

*“The water bottle refill stations are awesome because they actually have a water filter so that does improve the taste of the water” (School Personnel, P1).*

Stakeholders also noted barriers to drinking water, including students’ preferences for sugar sweetened beverages (SSB) and for chilled water temperature.

*“I think in general our kids are probably just not hydrated enough, you know, I see a lot of kids walking in the morning with drinks, but it's soda, its juice, it's sometimes coffee … It's like sugary Kool-Aid type things or Gatorade, and I mean it's not water*….” (Site Coordinator, P11).

One participant expressed how children are unaware of the benefits of drinking water.

*“Most of my work has been in elementary schools, so I would say that barriers [are] they aren't exposed to it as much, and they don't necessarily care about the health benefits*” (School Personnel, P22).

Stakeholders also noted that the novelty of the hydration stations impacted student hydration behaviors, reporting, “*It helps that the water refill stations are new so they are like, oh, I want to try it out”* (School Personnel, P12), but that poorly maintained water sources can deter students from using the hydration stations.

*“If the refillable water bottle station doesn't look clean or if you know there's something blocking it or there's something on top of it, it dissuades students from stopping to drink water from it*” (Wellness Leader, P14).

Several interviewees also reported that the hydration station filter status (green [new], yellow [nearing a need for replacement], and red [needs replacement]) impacts students’ use.

“… *What happens if the filter status is on the red dot? And who do we go to let them know about that? … Red dot doesn't mean a very good thing, so like I don't want to drink water from here,” and I kind of looked at the kids like, you know, I can't tell you that you're wrong*” (School Personnel, P25).

#### 3.1.3 Teachers’ role in student hydration

School personnel stakeholders (who were not teachers) emphasized the impact of teachers’ individual behaviors on shaping students’ perceptions of water.

“*We are not interested in telling teachers that they can't have their diet coke or whatever…we just ask that it be done not in front of students”* (Wellness Leader, P14).

Thus, school personnel could help prevent *“unintended messages”* about hydration by “*keeping [teacher] hydration choices out of view”* (School Personnel, P25). In contrast, others highlighted the importance of teachers’ role modeling healthy hydration, given their influence on students.

*“…I would put fruit infused water containers sometimes in the staff lounge, just during the week randomly, and email everyone and say, hey, there's cucumber water in the staff lounge today. Hope you enjoy it… hope was that they would take some, and then go back to their class and talk about it, and a lot of them did”* (School Personnel, P25).

“*I think teachers sharing that messaging is important because they are a big role model and a big piece of their kids’ lives”* (School Personnel, P22).

Stakeholders noted that students’ beliefs and attitudes about healthy hydration may be forged in the home environment.

### 3.2 Interpersonal level

#### 3.2.1 Home environment influences

Children may not be encouraged to drink water at home, in part due to the beverage habits and norms modeled by household members, which could shape beverage choices.

*“Maybe they're also not really hydrating properly at home, I don't think water is necessarily pushed in all homes as the best choice to drink...”* (School Personnel, P12).

*“…I actually feel like it has an important role because kids spend so much time at school and drinking water is a habit that many kids might not get from home…* (School Personnel, P25).

Additionally, beyond household beverage norms influences, limited access to clean water in the home was described as a barrier to students’ water consumption.

*“That autonomy, if they are thirsty or need to refill because I don't know what the water quality is like at home, so, at least at school there'll be able to get high-quality water and being able to stay hydrated throughout the day* (School Advocate, P15).

These home environment influences, including the beverage habits modeled by household members and limited access to clean water, highlight how factors outside of the school setting could shape students’ hydration behaviors before they even enter the classroom.

### 3.3 Institutional level

Stakeholders also described how the schools create cultures, either through formal and informal rules, which shape healthy hydration behaviors during the school day.

#### 3.3.1 Classroom management strategies

Specifically, stakeholders discussed how the classroom culture can influence hydration behaviors.

For example, strategies currently used in classrooms to encourage water intake such as *“hydration breaks*” (School Personnel, P12) during the school day:

*“So, I really try to make it one of my steps of the day to give the students multiple hydration breaks where we refill our water [bottles] and make sure that they are full and that they are drinking (water)”* (School Personnel, P12).

Additionally, stakeholders noted challenges with water bottle use in the classroom. One participant noted the following:

*“I think … they feel that the water bottles [are a] distraction because—and several teachers have said that they're [a] distraction—because the kids want to get up and go fill their water bottles—kids are playing with the water bottles…I know one teacher completely wiped-out water bottles.”* (School Advocate, P15)

Another common concern expressed was the need for frequent bathroom breaks (*“…disruption in day”;* School Personnel, P22).

*“I don't think teachers want to do a bunch of extra bathroom breaks during the day and I don't think that teachers really feel that's a good use of time to have students take the time to become more hydrated”* (School Personnel, P12).

Stakeholders also identified several strategies used by teachers to improve hydration behaviors, including, implementing structured bottle refill routines and assisting with reusable water bottle upkeep to facilitate consistent access to water throughout the school day.

“*We have a lot of good routines in place for the kids. They've really gotten used to, even if they're not directly next to a refill station, just quietly going and then coming straight back to class and they've been responsible with that...*” (School Personnel, P12).

#### 3.3.2 Access to resources

Stakeholders highlighted the accessibility of school hydration stations as an important facilitator of student water consumption. They noted, that the hydration stations were visible and in an accessible location, making it easier to encourage healthy hydration practices.

*“I’m on one end of the hallway and then there was actually a new water bottle refill station on the other end of the hallway recently… It's less than like 20 feet from every single classroom.... I’m really lucky because it's right next to my classroom door, so it's not a whole process. The kids can just quietly walk out refill their water bottle and come back”* (School Personnel, P12).

*“I love that they put more bottle fillers in the school. We used to only have the one by the cafeteria.”* (Site Coordinator, P11)

Despite the installation of hydration stations in schools, which improved student water access, several barriers were described by stakeholders, such as the limited access to water vessels needed to use the hydration stations.

*“It wasn't stated to keep these water bottles at school, it was sort of given the option to you can send all this stuff home, but then we have these new hydration systems. What are students supposed to refill them with? Where are extra water bottles coming from? And water bottles are not cheap”* (School Personnel, P12).

Some observed that school personnel navigated the ongoing challenges of inadequate resources by replacing lost or broken water bottles and encouraging students to keep their water bottles at school.

*“I saw one elementary school where the teacher allows them to keep the bottle there and they didn't take it home and they had their name on, and it was in their little cubby, so I love that, obviously”* (School Personnel, P13).

*“I know last year, a teacher … took them home every Friday to wash them which I thought was very kind and something I hadn't even thought about. So, like if you have classroom water bottles, how do they get clean? Which is just something else for teachers, if they have a classroom set, that they are in control of, which was really nice that that teacher used her Amazon wish list, and got the water bottles, and was washing them every week, but it also takes a lot on a human to do that”* (School Personnel, P22).

#### 3.3.3 Competing school priorities

Structural barriers, such as heavy teaching loads and testing demands coincide with students’ social challenges, including poverty, housing instability, and chronic absenteeism. Together, these factors create competing organizational priorities that could hinder efforts to addressing suboptimal hydration.

*“Homeroom teachers are so overburdened with many different things…homeroom teachers already have to, make sure that they're doing and performing in class, like hydration, unfortunately, would be at the very tail end of what they're thinking about”* (School Personnel, P25).

*“Testing scores determine a lot for the school, so I would say that is the number one focus”* (School Personnel, P12).

*“Our literacy is like pretty low, …We have kids who are experiencing homelessness. We have, a lot of families who are living in multifamily homes”* (Site coordinator, P10).

### 3.4 Community level

#### 3.4.1 Community influences

The community environment can influence students’ attitudes towards and access to beverage choices outside the school setting.

*“A lot of our kids don't have a grocery store that's near them, so most of their food shopping comes from, maybe gas stations or corner stores. They don't push water like we do. They're being exposed to all the different hydration choices that are out there, and that's just a big factor that we're trying to come back with”* (School Personnel, P25).

The availability of alternative beverage choices within students’ neighborhood environments, can reinforce individual (e.g., student motivation to drink water) and interpersonal level barriers (e.g., home environment) to healthy hydration behaviors.

### 3.5 Policy level

At the policy level, local regulatory policies, procedures, and guidelines, such as the school district’s wellness policy, serve to protect students’ health by increasing access to clean, potable, chilled water and promoting water friendly classroom practices.

#### 3.5.1 Awareness of current hydration policies

Stakeholders were asked to share perspectives on the school district’s hydration policy. Most were unaware of the district’s wellness policy.

*“… I don't know if there's something I miss, but I don't remember getting any information about a water policy, so I think that education piece needs to go there, and get everybody’s buy in and make it a school-wide effort… I think once everybody understands the policy, it'll make all the difference in the world with children hydration”* (Wellness leader, P26).

Stakeholders identified inconsistent communication about the hydration policy as key barrier to implementation:

*“There has been inconsistency, because, you know, different principals and teachers implement things differently or get information differently”* (Wellness leader, P16).

Furthermore, policy implementation has been challenging due to the varying levels of priority placed on school personnel.

*“I think the improvement will come in the knowledge of the fact that this policy exists; and that water access is now a priority, and steps need to be taken if it isn't, if water access is not available to all students”* (Wellness leader, P14).

Despite concerns and barriers related to the policy, stakeholders agreed that improving access to clean potable water and meeting students’ hydration needs during the school day are important.

*“I mean, the fact [is] that children need to be able to access water. So, I think the new policy is good”* (Site coordinator, P26).

Policy level barriers, such as limited awareness and inconsistent dissemination, often constrain facilitators (e.g., accessible hydration stations, availability of reusable water bottles) and reinforce existing barriers across multiple systemic levels of the SEM (individual, institutional, interpersonal, and community) demonstrating the need for feasible multi-level policy communication to support hydration efforts beyond the school setting.

## 4 Recommendations

Informed by stakeholders’ perspectives at each level of the SEM, recommendations for improving student hydration efforts (Table 2), including clear, targeted messaging for school personnel and families, securing teacher buy-in, and using multiple communication methods to engage parents and students.

**Table 2:** Stakeholders’ recommendations across levels of the socioecological model.

Level	Recommendations	Example quotation
Individual level	Enhance the taste of water	“... When we did the fruit infused water, just different ways to drink water that still makes it more flavorful and something that you are used to, but it is still water and not juice...so our, my goal was to show parents like, hey, I know your kid does not want to drink clean water, but here’s another option” (School Personnel, P22).
	Encourage role modeling among school personnel	“I think modeling, it is the biggest thing we can do, we can put up posters and do all of that, but most people do not read... I do learn from actions...making it accessible as is going to be the way for us to create that sustainability” (School Personnel, P13).
Interpersonal level	Implement water breaks	“Clear communication and allowing the students at their leisure, to be able to go and get water as needed. And having expectations around that so that kids do not take advantage of needing a water break every 5 min. (Wellness Leader, P26).
	Bathroom breaks	“So, making sure you are setting those boundaries from the very beginning. Kind of like what we talked about earlier, like [a] scheduled bathroom break is also bottle break for everybody. So, like, if you are feeling thirsty, like make sure you bring that with our bathroom breaks” (School Advocate, P15).
	Permit water bottle usage in the classroom	“Allow water bottles in the classroom... I mean like allowed them to do it like during class time, and that’s one of the benefits of having water bottles is that you know children are allowed to drink like during school…”(Wellness Leader, P26).
	Increase and standardize hydration messaging	“I think it would be how we communicate, or the school district communicate with students, currently, and I know a lot of that through the Google classroom so providing resources through their Google classroom platform may be helpful. I think the posting of educational pieces or things on social media is important as well” (Site Coordinator, P06).
Institutional level	Increase education on different benefits and preparation styles of water	“I would say, the education part about showing kids how they can play with their water with fruit: fruit infusion, mint, and urban fruit infusion of their water” (Wellness leader, P4).
	Establish standard practices for water bottle etiquette	“I think establishing some kind of rules for handling the water” (Wellness leader, P14).
	Resources for sustainability	“One of the things that I worry about with facility is, I can put the money into installing a hydration system, but are we going to have the money to replace the filters, or if something electrical goes wrong are we going to be able to fix it because we do not want it to be a vicious cycle, where we go back to where we did not have access at all because we cannot maintain what we are installing. For me, that’s always something I have to think about is how do I make this sustainable” (School Personnel, P13).
	Engage community partners in school-based hydration efforts	“… You know, bring something in that extra the after-school programs, the YMCA and [redacted] really partner together on after school programming. But that’s always a great place to start” (Site coordinator, P11).
Community level	Developing community partnerships	“[Need people] who champion things and get your school involved in healthy habits, champion those initiatives”; It’s been helpful to have partnerships. Some of our community partners who have assisted with helping, create a space where the kids understand how important it is…“Your wellness person used to be the person who was the champion those things and get your school involved and get people involved in doing healthy habits and champion those initiatives. So, they could do that with the kids” (School personnel, P13).
	Messaging to families	“…, the obvious one is the parents and the families, just making sure that we are sending clear-cut message, that we do value hydration” (School personnel, P25).
	Multiple communication methods	“I think that we have to use multiple ways…. whether it’s, text messaging…or Back to School nights or even some of the food distribution sites…, I would definitely encourage more than one method be used” (Wellness Leader, P16).
	Increase communication of the policy	“...So communicating the policy is important, even as a parent” (Site coordinator, P26).
Policy level	Provide necessary classroom and personnel resources to support effective policy implementation	“Teachers will like nod their head and smile, but then it’s not going to necessarily be followed, unless it’s actually, teachers have to buy into these things, you cannot just like say Okay, three times a day, you have to do this, because honestly, being a classroom teacher, we have so many things that we have to do every single day so adding another one thing to a lot of teachers is like, no I’m not going to do that (School Personnel, P12).”
	Provide clear communication on policy and related personnel action	“I think with any policy, it just takes time and people to talk about it. I think some things can be implemented and there’s just like so many moving pieces that it’s hard to know like what to do and what’s new this school year. I think making sure that the principles know” (School Personnel, P12).

To address inconsistent policy implementation, stakeholders recommended clear communication to improve district and school-wide understanding and allowing sufficient time for staff to adopt new practices. They also expressed the importance of financial resources to help manage costs associated with hydration stations maintenance.

Key individuals to sustaining a school-based healthy hydration program were identified. Most participants suggested that the physical education teachers and classroom teachers, or ideally, *“everybody to be on board”* (School Personnel, P25). The simplicity of a program was also highlighted as important to sustainability: “*if we are going to keep it sustainable, we just got to make it… we’ll call it, simple”* (School Personnel, P25).

## 5 Discussion

Several themes emerged across multiple levels of the SEM from the 15 semi-structured interviews with stakeholders. Stakeholders highlighted several addressable barriers (e.g., school-related competing priorities, access to drinking vessels) and facilitators (e.g., access to potable water, support from school personnel), to advance water equity through school-based interventions. To our knowledge, only one prior study has explored stakeholders’ perspectives on improving beverage intake among elementary school students; however, the study was limited to the perspectives of teachers (20). This study extends prior literature by capturing insight from diverse stakeholders (e.g., school personnel, community leaders) whom are internal and external to the school district, providing contextual insight into available needs and assets for intervention-based solutions. Findings reveal that multilevel interventions are needed to optimally address barriers and amplify facilitators of healthy hydration, as no single level of intervention alone is sufficient to advance water equity within this urban Title I school district.

### 5.1 Individual and interpersonal levels

At the individual level, stakeholders recognized water’s essential role in children’s physical and cognitive functioning. They believed that student motivators for using hydration stations included the novelty and appeal of the water sources. This is consistent with a prior school-based water initiative studies in elementary schools, which found that water education and promotion, along with “appealing” water sources, improved water access (23, 24). Installing more appealing water sources may further promote water intake in students (30). Cleanliness and water quality of the hydration station were also identified as key drivers of use, reinforcing the importance of timely maintenance, as noted in previous research (31). However, students’ preference for SSB was identified as a barrier to water consumption and should be considered in school-based hydration intervention initiatives. Additionally, stakeholders described how teachers’ hydration behaviors directly influence students, underscoring their role in student hydration during the school day (20, 32).

At the interpersonal level, beverage habits modeled by household members and perceptions of tap water safety at home also can further shape children’s beverage choices, particularly among students facing household water insecurity (33). These findings highlight the need to educate school personnel on the benefits of hydration and empower them to serve as role models. These findings also reinforce the importance of engaging household members alongside school personnel and stakeholders. Addressing broader influences, such as perceived water safety at home, can further support students’ beverage choices and foster long-term healthy hydration habits through school-based initiatives.

### 5.2 Institutional level

Schools, as institutions play a central role in shaping students’ hydration behaviors throughout the school day. However, findings suggest that this role is often constrained by competing priorities, resource gaps, and inconsistent classroom practices.

Stakeholders identified several barriers experienced by classroom teachers in supporting student hydration. Concerns included improper water bottle use in the classroom and the potential for increased bathroom breaks. These findings align with prior school-based hydration initiatives in elementary schools, in which classroom teachers reported restricting water bottles due to concerns about spills, distractions, and misuse (34). Concerns about increased bathroom breaks, highlight the need for appropriate policies and procedures that support normal voiding alongside adequate hydration. In one survey study of the 467 public elementary school teachers, 80% scheduled bathroom breaks, whereas only 40% allowed flexibility (35). Restrictive bathroom access during the school day may stem from teachers’ limited understanding of children’s elimination needs and can contribute to poor student toilet habits (e.g., dysfunctional voiding) (35). Supporting teachers in establishment of healthy hydration-promoting routines, along with appropriate education, might address these concerns.

Stakeholders expressed positive views about the district resources, including the installation of hydration stations, and were satisfied with their location, particularly when stations were near classrooms. Proximity of water stations was seen as key to improving the feasibility of hydrating students with less use of instructional time, representing an important institutional level facilitator. However, stakeholders indicated that additional resources are needed to support reusable water bottles availability, upkeep, and storage, as inconsistent access to vessels remained a barrier. Although, prior research suggests that alternative methods, such as positioning disposable cups near water sources, may efficiently increase student water consumption (36), concerns regarding cost and environmental impact often limit their viability as feasible solutions. Stakeholders suggest, including keeping water bottles at school and developing classroom policies related to their cleaning, storage, and use represent practical institutional level intervention targets that could address resource gaps while supporting sustainable hydration practices.

Collectively, these findings emphasize the importance of addressing institutional level barriers as potential intervention targets. Given the interplay across multiple levels of the SEM, institutional demands (e.g., academic priorities and resource constraints) that intersect with community level factors (e.g., poverty and housing instability), must be considered when designing an intervention to ensure acceptability and sustainability. For example, efforts must be cost-effective, low-burden, and responsive, to the unique institutional and community context (37).

### 5.3 Community level

At the community level of the SEM, environmental factors outside the schools setting were reported as potential influences on elementary school students’ hydration behaviors. Stakeholders’ views on community influences affecting student hydration are consistent with findings from previous studies. For example, frequent access to beverages in the home other than water has been associated with SSB consumption (38). The availability of SSB may also be influenced by neighborhood factors. One stakeholder described how some children primarily purchase groceries from convenience stores or gas stations. Children living in food insecure households demonstrate higher reliance on these sources, increasing their risk of SSB-related health issues, such as tooth decay (39).

Stakeholders also emphasized the value of partnering with community organizations already integrated in schools to enhance interventions efforts. Leveraging existing relationships may improve sustainability. Engaging school-based community partners can help to understand and address external factors that influence student hydration behaviors.

Findings highlight the importance of identifying factors within the school social environments, such as leveraging existing school-community partnerships and engaging neighborhood organizations. By creating environments that support rather than compete with healthy hydration behaviors, these consideration represent potential community level targets for advancing water equity (40).

### 5.4 Policy level

At the policy level of the SEM, while the district-level hydration policies provided a structural foundation for improving student hydration, most stakeholders reported general awareness of policy efforts, and noted school-level changes (e.g., newly installed hydration stations). However, many were unaware of specific changes, emphasizing a need for clearer policy dissemination and consistent, clear communication with school personnel. Previous research highlights the importance of an effective communication system among school staff and students, with an emphasis on bottom-up approaches. For example, Mukoro and colleagues recommend that school administrators employ multiple methods of communication to facilitate interpersonal communication, ensure consistency and clarity of messages, and to help build trust (41). Stakeholders similarly recommended using existing platforms to communicate hydration policies and programs.

### 5.5 Partnering with community advisory board members

The participatory approach employed in this study utilized an established CAB of key stakeholders from diverse roles who represented individuals who are nested within the different levels of the school context within the school district. CAB members were engaged throughout this work. For examples, they provided guidance on our research design (participant recruitment, i.e., stakeholders whose insight should be prioritized), the interview guide, and data interpretation. For example, CAB members expanded on findings related to classroom practices, by sharing their observations and lived experiences. By leveraging CAB members insights, their partnership helped to identify the most feasible initial intervention targets. For example, CAB members also provided important context on the broader community environment, including water equity challenges, factors that extend beyond the school setting. This reciprocal partnership between CAB members and our study team strengthened our work with stakeholders and enhanced the relevance and applicability of our findings for future school-based intervention development. This type of community engagement is consistent with best practices for community engaged research (16). Additionally, this approach is also consistent with evidence that academic-community partnerships bolster the development of sustainable, multilevel interventions that can build school capacity and foster a culture of health (14, 42).

## 6 Strengths and limitations

This study has several limitations. The use of convenience and snowball sampling methods may have introduced bias. Stakeholders’ narratives were not intended to be generalizable to the entire district or other school districts. Another important limitation is the lack of student perspectives. As the intended beneficiaries, we recognize student perspectives are essential to the development of effective school-based hydration interventions and have therefore engaged students in other aspects of our formative work. For example, we have partnered with elementary school student ambassadors to co-design student-led healthy hydration messaging and marketing materials for their schools. Although, parents were not directly targeted in this study given the focus on interventions in the school environment, they play a critical role in shaping children’s hydration behaviors and attitudes toward drinking water at school. Notably, our CAB includes parents and students, and their perspectives were reflected in the interpretation of the current findings.

Strengths of this study include engagement of our CAB in all aspects of this work from study inception through implementation and data interpretation. Our community-engaged qualitative approach yielded rich, emerging themes regarding elementary schools’ hydration landscape. Additionally, we explored a broad range of factors across the SEM, including individual perspectives, school practices, and policies. In these ways, we enhanced our understanding of the hydration landscape within a local school district.

## 7 Conclusion

Advancing water equity within the school setting requires appropriate community resources; collaboration among community partners, key liaisons and families; clear communication; and consistent procedures. Guided by the SEM, stakeholder insights provide timely and valuable information that can bolster efforts to support federally mandated policies designed to increase water access in schools, while identifying multilevel environmental factors that must be addressed to translate policy intent into practice. Importantly, findings underscore that improving student hydration requires moving beyond individual level targets to address the broader environmental factors across the individual, interpersonal, institutional, community, and policy levels that shape student hydration behaviors.

The key contributions of this study were the actionable recommendations provided by our stakeholders with indirect and direct roles in an urban Title 1 elementary school district, highlighting the voice and input of those most impacted and knowledgeable about their community’s strengths and needs. Consideration of strengths (e.g., availability and appeal of water bottle refill stations; teacher strategies for managing water bottles at schools), alongside challenges, including student, school personnel, home and neighborhood influences, inconsistent communication, limited resources, and competing school priorities, can enhance the relevance, feasibility, and ultimately effectiveness of hydration efforts within Title I elementary schools.

## Data Availability

The datasets presented in this article are not readily available because this research involved qualitative data that was generated from individual semi-structured interviews. We then pulled data from these conversations and included them in our article, including the quotes. However, the raw qualitative data is not able to be shared since it possesses identifiable information from the subjects. Requests to access the datasets should be directed to tatumk@vcu.edu.

## Appendix A

Interview guide domains and questions

1. What does healthy hydration mean to you?
2. Please share your thoughts about why, if at all, hydration (i.e., drinking water) is important for children?
3. Please describe your role and any involvement with RPS?
4. Probe: How does your position play a role, if at all, in student health?

Student hydration (behavior)

1. From your perspective, what are some factors that might help improve student hydration?
2. What are some things that make supporting student hydration challenging? From your perspective, what are some barriers to student hydration?

Current hydration policies

1. Were you aware of this policy change?
2. What are your thoughts on the new policy?

Improving student hydration

1. What strategies would help improve student hydration?
2. From your perspective, who do you think would be a critical partner at your school to help launch a new program to improve hydration in RPS students?
